# Histopathological and biochemical investigations of protective role of honey in rats with experimental aflatoxicosis

**DOI:** 10.1186/s12906-016-1217-7

**Published:** 2016-07-21

**Authors:** Turan Yaman, Zabit Yener, Ismail Celik

**Affiliations:** Department of Pathology, Faculty of Veterinary Medicine, Yuzuncu Yil University, Van, Turkey; Department of Biology, Faculty of Sciences and Letters, Yuzuncu Yil University, Van, Turkey

**Keywords:** Aflatoxicosis, Honey, Histopathology, Malondialdehyde, Antioxidant, Rat

## Abstract

**Background:**

Natural honey (honey) is considered as a part of traditional medicine all over the world. It has both antimicrobial and antioxidant properties, useful in stimulation of wounds and burns healing and gastric ulcers treatment. The aim of this study, for the first time, was to investigate the antioxidant properties and protective role of honey against carcinogen chemical aflatoxin (AF) exposure in rats, which were evaluated by histopathological changes in liver and kidney, measuring level of serum marker enzymes [aspartate aminotransferase (AST), alanin aminotransferase (ALT), gamma glutamil transpeptidase (GGT)], antioxidant defense systems [Reduced glutathione (GSH), glutathione reductase (GR), superoxide dismutase (SOD), glutathione-S-transferase (GST) and catalase (CAT)], and lipid peroxidation content in liver, erythrocyte, brain, kidney, heart and lungs.

**Methods:**

Eighteen healthy Sprague–Dawley rats were randomly allocated into three experimental groups: A (Control), B (AF-treated) and C (AF + honey-treated). While rats in group A were fed with a diet without AF, B, and C groups received 25 μg of AF/rat/day, where C group additionally received 1 mL/kg of honey by gavage for 90 days.

**Results:**

At the end of the 90-day experimental period, we found that the honey supplementation decreased the lipid peroxidation and the levels of enzyme associated with liver damage, increased enzymatic and non-enzymatic antioxidants in the AF + honey-treated rats. Hepatoprotective and nephroprotective effects of honey is further substantiated by showing almost normal histological architecture in AF + honey-treated group, compared to degenerative changes in the liver and kidney of AF-treated rats. Additionally, honey supplementation ameliorated antioxidant defens systems and lipid peroxidation in content in other tissues of AF + honey treated rats.

**Conclusion:**

The present study indicates that honey has a hepatoprotective and nephroprotective effect in rats with experimental aflatoxicosis due to its antioxidant activity.

## Background

Aflatoxins (AF), a group of mycotoxins which are produced as secondary metabolites by *Aspergillus flavus* and *Aspergillus parasiticus*, are common contaminants in a wide variety of food and feed products [1]. These toxins are produced by fungal activity during production, harvest, transportation, storage and food processing [2]. Aflatoxin B_1_ (AFB_1_) has teratogenic, carcinogenic, mutagenic and immunosupressive effects in both humans and animals, which is the most important and the potent hepatotoxic and hepatocarcinogenic agent [3] and has been classified as a carcinogenic agent to humans by the International Agency for Research on Cancer [4].

Oxidative stress is a common mechanism in initiation and progression of hepatic damage in aflatoxicosis [3]. When animals or humans consume the foods containing aflatoxins, AFB_1_ is metabolized in liver, producing highly reactive chemical intermediaries which cause cell damage, free radical production, and lipid peroxidation [5, 6]. Two forms of aflatoxicosis have been identified: the first is acute severe intoxication, which results in direct liver damage and subsequent illness or death, and the second is chronic subsymptomatic exposure. Symptoms of severe aflatoxicosis include; hemorrhagic necrosis of liver, bile duct proliferation, edema, and lethargy. In farm and laboratory animals, chronic exposure to aflatoxins compromises immunity and interferes with protein metabolism and multiple micronutrients those are critical to health [7].

Natural honey (honey) is a remarkable liquid prepared by bees from the nectar of many plants. All over the world, it is considered as a part of traditional medicine, especially in gastrointestinal disorders. There are few reports about the effectiveness of honey in gastric ulcers or gastrointestinal disorders in humans [8, 9]. Studies have shown that honey has nephroprotective [10, 11], antibacterial, anti-inflammatory and antioxidant properties [12], useful in stimulation of diabetic wounds and burns healing [13] and gastric ulcers treatment [14]. The carbohydrates are the main constituents, comprising about 95 % of the honey dry weight. Beyond carbohydrates, honey contains numerous compounds such as organic acids, proteins, amino acids, minerals, polyphenols, vitamins and aroma compounds [15]. Additionally, honey has rich flavonoid components [16].

Free radicals lead to oxidative damage in many molecules, such as lipids, proteins and nucleic acids. Many complications have been attributed to oxidative damage, including atherosclerosis, aging, and cancerous diseases. Antioxidant-rich foods that are rich in flavonoids have protective potentials against these ailments [17].

There is no study on chemoprotective effects and antioxidant roles of honey against AF-induced tissue damages in rats. Therefore, with the present study we aimed to examine the protective effect of honey against AF-induced liver and kidney injury by assessing histopathology and biochemical parameters including liver enzymes, lipid peroxidation and antioxidant defense systems in erythrocyte, brain, liver, kidney, heart and lungs of rats.

## Methods

### Chemicals

Thiobarbituric acid (TBA), butylated hydroxytoluene (BHT), trichloroacetic acid (TCA), ethylenediaminetetraacetic acid (EDTA), reduced glutathione (GSH), metphosphoric acid, 5,5’dithiobis-(2-nitrobenzoic acid) (DTNB), trihydroxymethyl aminomethane (Tris), 1-chloro-2,4-dinitrobenzene (CDNB), oxidized glutathione (GSSG), β-Nicotinamide adenine dinucleotide phosphate (reduced) (NADPH), potassium dihydrogen phosphate (KH2PO4), hydrogen peroxide (H2O2) and sodium chloride (NaCl) of technical grade used in this study were supplied by Sigma Chemical Co. (St. Louis, MO, USA). Kits for antioxidant enzymes analysis were supplied by Randox Laboratories Ltd. The honey sample of East Anatolia region was purchased from a local honey store in Van, Turkey.

### Aflatoxin

The AF was produced (in the Department of Pharmacology and Toxicology, Faculty of Veterinary Medicine, University of Selçuk, Konya; Turkey) from Aspergillus parasitcus NRRL 2999 culture (USDA, Agricultural Research Service, Peoria, IL, USA) via fermentation of rice by the method of Shotwell et al [18] with minor modifications by Oguz [19]. Successfully fermented rice was then steamed to kill the fungus, dried and ground to a fine powder. The AF content in rice powder was analyzed by the method of Shotwell et al [18] and measured on a thin layer chromatography (TLC) fluorometric densitometer (Camag-III, Basel, Switzerland) on TLC spots. The AF within the rice powder consisted of 72.51% AFB_1_, 14.05% AFB_2_, 9.78% AFG1 and 3.66% AFG2 based on total AF in the ground rice powder (detection limit: 1 mg AF kg–1 rice powder; recovery of the extraction method: 92%). The rice powder was incorporated into the basal diet to provide the required amount of feed, 25 μg of AF/rat/day.

### Animals

A total of 18 Male Sprague–Dawley rats, weighing 300–350 g, on average, at 5.5-month-old were provided by the animal house of the Medical School of Yuzuncu Yil University. Animals were housed at 20 ± 2 °C in daily light/dark cycle. All animals were kept in stainless cages and fed with wheat-soybean-meal-based diet. Water and feed were offered ad libitum. The animals received humane care according to the criteria outlined in the ‘Guide for the Care and Use of Laboratory Animals’ prepared by the National Academy of Science and published by the National Institutes of Health. The ethic regulations have been followed in accordance with The National and Institutional guidelines for the protection of animal welfare during experiments. Ethical approval was obtained from the Animal Experiments Local Ethics Committee of Yuzuncu Yil University.

### Experimental model

A total of 18 rats were randomly allocated into three experimental groups: Group A (Control, with the basal diet without AF), group B (a diet with AF), and group C (a diet with AF + honey), each containing 6 animals. The rats in group B and group C received 25 μg of AF/rat/day. The rats in group C also received 1 mL/kg of honey by gavage for 90 days. The experiment lasted 90 days. Animals were weighed on the 0th, 30th, 60th and 90th days of the experiment. At the end of the 90-days experimental period, after the rats were anesthetized by inhalation of diethyl ether, blood samples were obtained by cardiac puncture using syringe for the determination of serum enzyme levels and biochemical analysis. Tissue samples were also obtained after sacrifying the animals. For analysis of serum enzyme levels, blood samples were put immediately into ice-chilled siliconized disposable glass tubes. Serum samples were obtained by centrifuging blood samples at 4000 × g for 15 min. at 4 °C, and enzyme levels were measured in the serum samples. For biochemical analysis, blood samples were put immediately into silicon disposable glass tubes with EDTA as an anticoagulant, which were centrifuged at 4000 × g for 15 min at 4 °C and erythrocyte pellets were obtained. Then, pellets were washed three times with physiological saline (0.9 % NaCl). The levels of GSH in erythrocytes and tissues were measured immediately after the animals were sacrificed because of tremendous loss of GSH when delayed. The levels of GSH and MDA and the activities of SOD, GR, CAT and GST in erythrocytes were measured in the pellets.

Samples of the liver tissues were dissected and put in Petri dishes. After washing the tissues with physiological saline (0.9 % NaCl), samples were taken and kept at -78 °C until analysis. Tissues were homogenized for 5 min in 50 mM ice-cold KH2PO4 solution (1:5 w/v) using a glass-porcelain homogenizer (20 KHz frequency ultrasonic, Jencons Scientific Co.) for 5 min and then centrifuged at 7000 × g for 15 min. All processes were carried out at 4 °C. Supernatants and homogenates were used to determine for measurements of enzymatic and non-enzymatic antioxidants and MDA.

### Biochemical analysis

The concentrations of MDA in erythrocyte and tissues were determined using the method described by Jain et al [20] based on TBA reactivity. GSH concentrations in erythrocyte and tissues were measured using the method described by Beutler et al. [21]. CAT (EC 1.11.1.6) activity was determined using the method described by Beutler [22]. GST (EC 2.5.1.18) was assayed at 25 ^o^C spectrophotometrically by following the conjugation of glutathione with 1-chloro-2, 4-dinitrobenzene (CDNB) at 340 nm as described by Mannervik and Guthenberg [23]. GR (EC 1.6.4.2) activity was assayed at 37 °C and 340 nm by following the oxidation of NADPH by GSSG [22]. SOD (EC 1.15.1.1) activity was measured at 505 nm and 37 °C and calculated using inhibition percentage of formazan dye formation [24].

### Measurement of enzyme levels

Serum enzyme activities (AST (EC 2.6.1.1), ALT (E.C 2.6.1.2), and GGT (E.C. 2.3.2.2)) were measured by an auto analyzer (BM/HITACHI-911), using kits (DPC; Diagnostic Products Corporation, USA).

### Analysis of data

All data were expressed as mean ± standard deviation (SD). The statistical analyses were made using Minitab 13 for windows packet program. One way ANOVA statistical test was used to determine the differences between means of the treatments and the control group accepting the significance level at p ≤ 0.05. The differences among the pathological changes were also determined by chi-square test. Statements of statistical significance are based on *P* <0.01.

### Histopathological examination

After taken from rats following scarification, liver and kidney tissue samples were fixed in 10 % neutralized formaldehyde, embedded in paraffin wax and then stained with hematoxylin and eosin. In addition, masson-trichrome and sudan black B methods were used specifically to stain collagen tissues and to demonstrate fatty changes, respectively.

Microscopically hepatocellular degeneration was graded in three ways; Slight: Mild hepatocellular swelling and fatty changes localized only around the periacinar areas. Moderate: Clear hepatocellular swelling in both periacinar and midzonal areas. Severe: Diffuse and severe hepatocellular swelling, cytoplasmic pallor and rupture [25].

## Results

### Effects on body weights

During the 90-days experimental period, none of the animals showed any clinical signs of illness and there were no mortality in any groups. Body weights of the rats significantly decreased (*P* < 0.05) in AF-treated group, and increased (*P* < 0.05) in control and AF + honey-treated groups. Effects of AF and honey on body weights of rats were given in Table 1

**Table 1:** Effects of AF and honey on body weight of rats

Parameters	A (Control)		B (AF-treated)		C (AF + Honey-treated)	
	Mean ± SD		Mean ± SD		Mean ± SD	
Body weight (g)	Beginning	Final	Beginning	Final	Beginning	Final
	322 ± 2.9	335 ± 1.8^a^	325 ± 2.1	312 ± 1.9^b^	323 ± 1.6	334 ± 2.3^a^

Each value represents the Mean ± SD^a^: significantly different from control, ^b^: significantly different from group B at *p* < 0.05 (One way ANOVA)

### Effects on biochemical parameters

Levels of liver enzymes (AST, ALT and GGT) of control, AF-treated and AF + honey-treated groups are summarized in Table 2. The levels of these enzymes were significantly elevated in AF-treated group in comparison to control group (*p* < 0.05). Whereas, the levels of these enzymes were markedly dropped in AF + honey-treated group with respect to AF-treated group. Besides, there were no significant differences in AST, ALT and GGT levels between control and AF + honey-treated groups (Table 2).

**Table 2:** Effects of AF and honey on serum enzyme levels of rats

Parameters	A (Control)	B (AF-treated)	C (AF + Honey-treated)
	Mean ± SD	Mean ± SD	Mean ± SD
AST (U/L)	161.5 ± 39.2	248.5 ± 66.6^a^	183.3 ± 32.4^b^
ALT (U/L)	43.0 ± 9.1	115.5 ± 27.7^a^	57.2 ± 7.3^b^
GGT (U/L)	0.1 ± 0.04	2.8 ± 1.2^a^	0.84 ± 0.08^ab^

Each value represents the Mean ± SD. ^a^: significantly different from control, ^b^: significantly different from B group rats at *p* < 0.05 (One way ANOVA)

The level of GSH and MDA, the activities of CAT, GST, GR and SOD enzymes in tissue samples taken from liver, lung, kidney, heart and brain of all groups are given in Table 3. As it can be viewed in the Table, prominent changes were determined. For example, there were significant decreases in the activities of CAT, GR and SOD in AF-treated group, when compared to control group (*p* < 0.05). As compared with AF-treated group, honey supplementation significantly ameliorated the activities of the antioxidant enzymes (CAT, GR and SOD) in some tissues. Furthermore, there were no significant differences in the levels of these antioxidant enzymes between control and AF + honey-treated groups (*p* < 0.05).

**Table 3:** Effects of AF and honey on antioxidant defense system and MDA contents in various tissues of rats

Tissue	Parameters	A (Control)	B (AF-treated)	C(AF + Honey-treated)
		Mean ± SD	Mean ± SD	Mean ± SD
Erythrocyte	GSH mg/dl	39.5 ± 0.84	38,8 ± 1,2	43,8 ± 1,5
	MDA nmol/ml	0,39 ± 0,08	0,69 ± 0,2^a^	0,49 ± 0,1^ab^
	CAT U/ml	461.1 ± 40.4	112.6 ± 12.1^a^	401.4 ± 147.1^b^
	GST U/ml	0,62 ± 0,16	0,75 ± 0,39	0,35 ± 0,21
	GR U/ml	0.37 ± 0.12	0.7 ± 0.34^a^	0.85 ± 0.29^a^
	SOD U/ml	428,6 ± 10,5	443,5 ± 3,5	436,5 ± 13,5
Brain	GSH mg/g	28.4 ± 4.4	32.5 ± 5.4	33.7 ± 3.8
	MDA nmol/g	34.1 ± 2.4	38.1 ± 3.4	38.3 ± 4.7
	CAT U/g	54.9 ± 13.3	52.3 ± 21.5	55.6 ± 12.2
	GST U/g	2.6 ± 0.3	2.6 ± 0,3	2.8 ± 0,5
	GR U/g	0.94 ± 0.2	0.83 ± 0.14	0.73 ± 0.07
	SOD U/g	1511.7 ± 115.2	1605.1 ± 55.3	1631.6 ± 55.3
Liver	GSH mg/g	27,5 ± 2,1	21,8 ± 0,5^a^	24,6 ± 1,5
	MDA nmol/g	35,4 ± 3,04	73,7 ± 13,8^a^	49,1 ± 8,1^ab^
	CAT U/g	79,7.4 ± 13.2	30.8 ± 13.7^a^	66.3 ± 17.6^b^
	GST U/g	8,8 ± 1,2	8,7 ± 2,7	8,5 ± 1,6
	GR U/ml	1.34 ± 0.1	0.85 ± 0.11^a^	0.80 ± 0.19^a^
	SOD U/g	1879,7 ± 36,8	1451,9 ± 275^a^	1582,5 ± 90^a^
Kidney	GSH mg/g	46.5 ± 0.6	45.7 ± 1.9	45.7 ± 1.8
	MDA nmol/g	6.3 ± 2.5	72.9 ± 43.8^a^	11.9 ± 3.1^ab^
	CAT U/g	24.1 ± 11.9	24.8 ± 14.7	20.7 ± 2.8
	GST U/g	6.5 ± 1.1	5.3 ± 0,3	4.6 ± 0,4
	GR U/g	0.37 ± 0.1	0.26 ± 0.1	0.3 ± 0.1
	SOD U/g	1504.7 ± 161.8	1485.6 ± 303.6	1468.3 ± 250.7
Heart	GSH mg/g	36.4 ± 0.9	39.6 ± 2.3	41.1 ± 1.7
	MDA nmol/g	7.12 ± 1.2	21.8 ± 1.2^a^	8.3 ± 3.1^b^
	CAT U/g	22.8 ± 1.5	16.7 ± 4.1	16.7 ± 6.3
	GST U/g	2.5 ± 0.19	2.8 ± 0,2	2.17 ± 0,3
	GR U/g	1.2 ± 0.16	1.15 ± 0.06	1.15 ± 0.06
	SOD U/g	1349.3 ± 69.4	1471.3 ± 78.5	1467.9 ± 8.2
Lungs	GSH mg/g	39.9 ± 4.8	27.8 ± 3.5^a^	39.4 ± 3.2
	MDA nmol/g	11.8 ± 2.8	73.4 ± 11.8^a^	42.3 ± 6.1^ab^
	CAT U/g	43.5 ± 14.2	37.5 ± 17.6	37.5 ± 22.3
	GST U/g	6.2 ± 1.3	5.7 ± 1,2	3.3 ± 0,5
	GR U/g	1.3 ± 0.14	1.5 ± 0.08	1.2 ± 0.2
	SOD U/g	1879.8 ± 36.8	1643.6 ± 112.1^a^	1582.6 ± 89.9^a^

Each value represents the Mean ± SD. ^a^: significantly different from control, ^b^: significantly different from B group rats at *p* < 0.05 (One way ANOVA)

MDA levels of tissue and erythrocytes in rats were significantly higher in AF-treated group and AF + honey-treated group in comparison to the control group (*p* <0.05). However, MDA levels of tissue and erythrocytes in AF + honey-treated group were considerably, in most cases significantly, lower than those of the AF-treated group (*p* < 0.05). This result indicating restorative effects of honey on the MDA levels of tissue and erythrocytes (Table 3).

### Histopathological findings

No gross lesions were observed in the hepatic structure of rats in control and AF + honey-treated groups. However, the livers of the AF-treated group were mild pale and swollen. The gross and histopathological lesions and the number of affected animals are summarized in Table 4, in which the severity of the changes was classified from slight to severe.

**Table 4:** Effects of AF and honey on the Liver and kidney structure

Changes/lesions in liver	A (Control) Mean ± SD	B (AF-treated) Mean ± SD	C (AF+Honey-treated) Mean ± SD	*P* values
1. Enlargement and palaness	-/6^b^	2/6^a^	-/6^b^	*
slight	*	2	*	
Moderate	*	*	*	
Severe	*	*	*	
2. Hydrolic degeneration/ or fatty changes	-/6^b^	6/6^a^	6/6^c^	
Slight	*	*	4	**
Moderate	*	2	2	
Severe	*	4	*	
3. Dysplastic hepatocytes	-/b^b^	6/6^a^	4/6^c^	**
Slight	*	*	2	
Moderate	*	3	2	
Severe	*	3	*	
4. Bile-duct proliferation	-/6^b^	5/6^a^	2/6^c^	**
5. Periportal fibrosis	-/6^b^	6/6^a^	1/6^b^	*
Slight	*	2	1	
Moderate	*	2	*	
Severe	*	2	*	
6. Degeneration and necrosis in renal tubule epithelial cells	-/6^b^	6/6^a^	2/6^c^	*
Slight	*	2	2	
Moderate	*	4	*	
Severe	*	*	*	
7. Megalocytosis in renal tubule epithelial cells	-/6^b^	2/6^a^	-/6^b^	
Slight	*	2	*	
Moderate	*	*	*	
Severe	*	*	*	

The values present the number of rats showing change/number of rats examined in each treatment group. Values with different letters (a, b and c) in same row are significantly different (*P* <0.01), according to the Chi-Square tests.

*P >0.05

**P <0.01

**Fig. 1:**
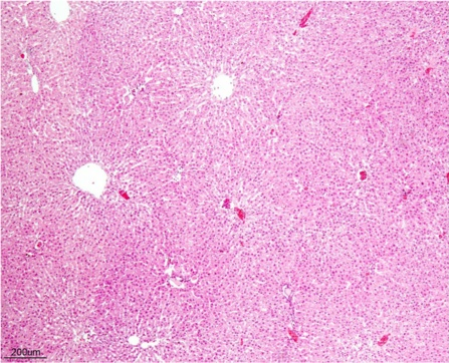
Control (Grup A): Normal histological appearance of the liver. H. E. Bar = 200 μ

**Fig. 2:**
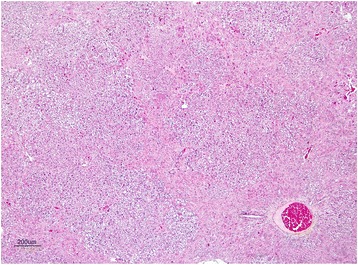
AF-treated group: Large degenerative and necrotic areas in liver parenchyme. H. E. Bar = 200 μ

**Fig. 3:**
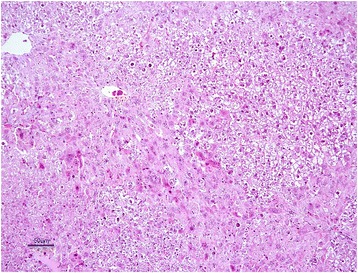
AF-treated group: Degeneration and necrosis of hepatocytes. H. E. Bar = 50 μ

**Fig. 4:**
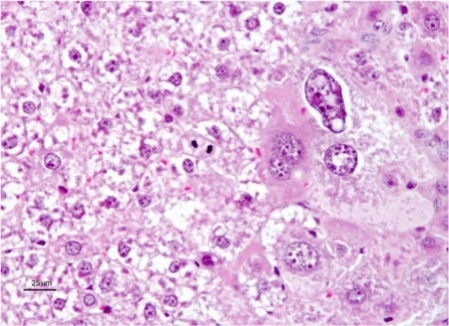
AF-treated group: Dysplastic hepatocytes, nucleus of which demonstrates anenlarged, hyperchromatic and pleomorphic appearence with a coarse chromatin pattern, their cytoplasms distincly large (megalocytosis) H. E. Bar = 25 μ

**Fig 5:**
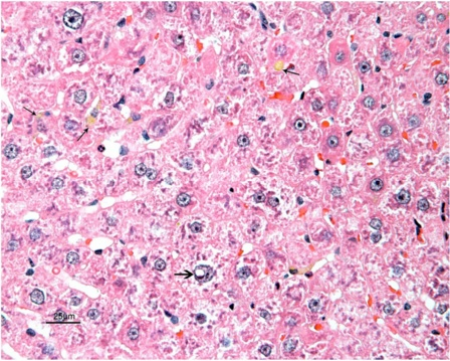
AF-treated group: Intrahepatic cholestasis (thin arrows) and vesicular nuclei (thick arrow). H. E. Bar = 25 μ

**Fig 6:**
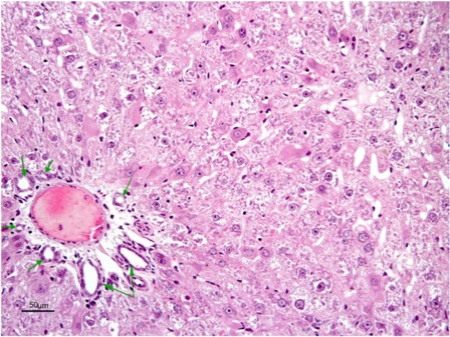
AF-treated group: Bile-duct hyperplasia (arrows) H. E. Bar = 50 μ

The livers of rats from control group had normal histological appearance (Fig. 1). Marked histopathological lesions were consistently observed in livers of all rats from AF-treated group. Inspections of the liver sections showed severe histopathological changes in all rats of this group. Predominantly, hydropic degeneration, necrotic changes and dysplastic changes in hepatocytes were detected. We noted that cloudy swelling or vacuolar-hydropic degenerative hepatocytes had located particularly in the periacinar and intermediate regions of the liver lobules and characterized with large foci of hepatocytes with granulated cytoplasm. Some hepatocytes in the periportal regions had moderate to severe cytoplasmic vacuolation, indicating fatty change. This condition was confirmed with Sudan black B stain. Partially normal-looking hepatocytes were encountered in the parenchyma resided usually in the periportal areas and among the degenerative foci. But often focal necrosis were formed in this regions (Figs. 2 and 3). In these areas, the nuclei of some cells were picnotic or karyorrhectic, and their cytoplasms were either lysed or eosinophilic. Furthermore, the nuclei of the hepatocytes were of different sizes and showed varying degrees of staining; while hepatocytes possessed two nuclei, others contained 2-4 prominent nuclei. The cytoplasm and nuclei of some degenerative hepatocytes were noted to be very large and abnormally shaped (megalocytosis) (Fig. 4). Moreover, the vesicular shaped nuclei of some degenerated hepatocytes showed marginal hyperchromasia and they were empty. Additionally, mitotic figures were encountered in some degenerative hepatocytes. The arrangement of remark cords was impaired owing to severe degenerations and the compressive focal atrophy was formed in the parenchyma around the severe degenerative cell foci. Hyperemia in some veins and sinusoids, increase of the number perisinusoidal cells, and intrahepatic cholestasis (Fig. 5) were also noticed. Likewise, epithelial hyperplasia in the bile ducts and an increase in the number of bile ducts, and focal mononuclear cell infiltration in the portal area were observed in five of the six rats in group B (AF-treated group) (Fig. 6). Although an increase in connective tissue was observed in portal gaps, there were no proliferations of the connective tissue spreading to the parenchyma from portal gaps.

**Fig 7:**
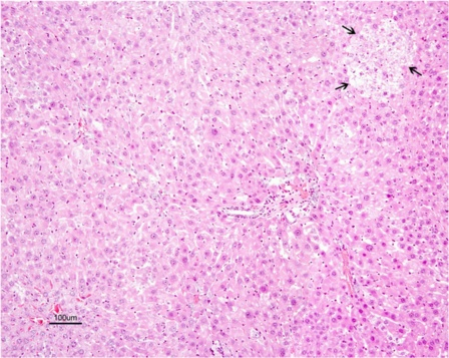
AF + honey treated group: Slightly degenerative and dysplastic hepatocytes in parenchyme and foci of degenerative cells (arrows). H. E. Bar = 100 μ

Alterations were also noted in the livers of the rats in AF + honey treated group but these changes were significantly less when compared to the rats in the AF-treated group. Examinations of livers in this group showed slight hydropic degenerations and necrotic changes. Lesions were localized in some liver lobules in the form of degenerative cell foci with fewer cells. However, the architectures of lobules were intact (Fig. 7). Moreover, some apoptotic and dysplastic cells as well as megalocytic hepatocytes were rarely encountered. Proliferation in the bile ducts in two of the six rats in this group and light periportal fibrosis in one rat were also noted.

**Fig 8:**
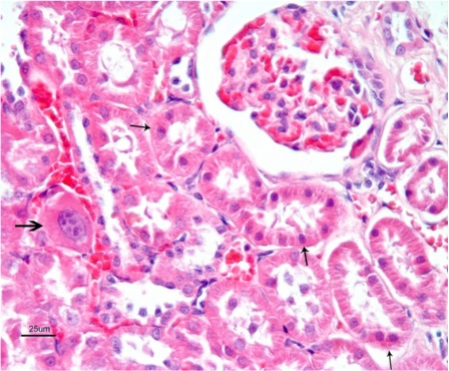
AF-treated group: Necrosis in renal proximal tubular epithelium (thin arrows) and megalosit tubule epithelial cells (thick arrow) with capillary hyperemia. H. E. Bar = 25 μ

Kidney sections of control rats showed normal histological appearance of the glomeruli, and renal tubules in the cortical and medullary portions. Marked histopathological changes were observed in kidney of rats from AF-treated group. These changes; degeneration and coagulation necrosis of proximal tubule epithelial cells, hyperemia in arterioles, glomerular and interstitial capillaries and hyaline casts in some of the tubule lumens. Furthermore, megalocytic cells were seen in tubular epithelium (Fig. 8).

**Fig 9:**
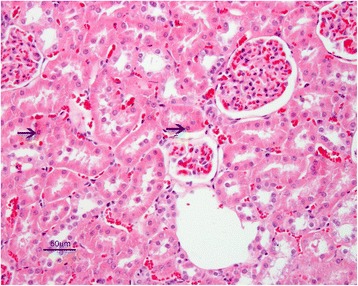
AF + honey treated group: Slight degenerative and necrotic changes in some tubule epithelial cells (arrows). H.E. Bar = 50 μ

In AF + honey-treated group, the kidney tissue exhibited normal histological appearance similar to the control group. However, slight degenerative and necrotic changes were seen in some tubule epithelial cells. There was also presence of capillary hyperemia (Fig. 9). Histopathological lesions and the number of affected animals are summarized in Table 4, in which the severity of the changes was classified from slight to severe.

## Discussion

The present study, for the first time, demonstrated that the treatment with honey effectively protected the rat against AF-induced hepatotoxicity, as evidenced by decreased AST, ALT and GGT levels and hepatic lipid peroxidation and elevated antioxidants levels. These protective effects of honey against AF-induced hepatotoxicity were also confirmed by histopathological investigation.

Previous studies indicate that body weights of the rats receiving AFB_1_ decreases. Body weights in mice are reported to be reduced by a range from 37 to 55 % depending on dose and duration of AFB_1_ exposure [26]. Aflatoxicosis is shown to cause hypoproteinemia by preventing protein synthesis; thereby, causing loss of body weight [27]. Results in the present study also demonstrated that the rats fed with AF had less body weight at the end of the 90-days experimental period, as compared with control group, whereas, there were increases in body weight at the end of the 90-days experimental period in the rats that received AF + honey and control rats.

The known histopathological and biochemical findings of the AFB_1_ toxicity in rats were also observed in the present study. The hepatotoxic effects of AF have been well documented in a variety of animal species [28, 29]. The main mechanism by which aflatoxin induces hepatotoxicity is formation of reactive oxygen species (ROS) and consequent peroxidative damage [30]. AFB_1_ is shown to induce lipid peroxidation in cells via inducing A2 and thereby disrupts the integrity of cell membranes [31]. AFB_1_ can also stimulate formation of ROS [32], occurrence of lipid peroxidation, and generation of 8-hydroxydeoxyguanosine [33]. Considering the role of ROS in chemically induced carcinogenesis, the ability of AFB_1_ to induce oxidative damage to cells and DNA may, in addition to the formation of AFB_1_–DNA adducts, play an important role in AFB_1_ carcinogenicity [34]. Generation of epoxide derivatives produced via aflatoxin metabolism, which depend on especially hepatic microsomal cytochrome P-450, is suggested to play a crucial role on the emergence of AFB_1_ toxicity and its carcinogenic effects [35]. Moreover, these epoxide metabolites are reported to disturb cell integrity through binding to macromolecules such as nucleoproteins and nucleic acids, and consequently inhibiting enzyme and protein synthesis [35, 36]. These enzyme systems (hepatic microsomal cytochrome P-450) are revealed to be localized at the highest concentration in the hepatocytes residing in the vicinity of periacinar areas of the liver [37]. In our study, histopathological changes such as cloudy swelling, hydropic degeneration, and focal necrosis were noted primarily in the periacinar regions and intermediate regions of liver lobules of the animals in treatment groups, especially AF-treated group. Primarily periacinar and secondarily intermediate localizations of liver lesions in aflatoxicosis are also emphasized by other researchers [38, 39]. Moreover, hepatocellular degeneration in aflatoxicosis is graded in three ways: 1) mild lesions characterized with hepatocellular swelling due to hydropic degeneration and fatty changes, 2) moderate lesions distinguished with clear hepatocellular swelling confined around the periacinar and midzonal areas, and 3) severe lesions typified with diffused-severe hepatocellular swelling, cytoplasmic pallor and rupture [25]. In the current study, we noted distribution of similar lesions that are presented in Table 4.

Aflatoxicosis is shown to cause several microscopic changes in the liver, i.e., development of cytomegaly, formation of evident nucleolus, appearance of marginal hyperchromasia, and occurrence of hepatocytes with two nuclei [38]. Likewise, in the present study we noted similar changes in hepatocytes particularly in AF-treated group and less in honey + AF-treated group. This observation might be probably related to increased RNA and DNA synthesis owing to aflatoxin-associated suppression of cell division as stated by the study of Gabliks et al. [40].

Several studies indicate that aflatoxicosis causes epithelial hyperplasia and proliferation in the liver bile ducts [41, 42]. The severetiy of these changes increases as a function of dose of AFB_1_ and its duration of availability. Besides, aflatoxicosis causes necrotic changes in the liver, which is one of the liver reactions to any damages to the liver [38]. In this study, epithelial proliferation and hyperplasia were observed in five cases in the AF-treated group and in two cases in AF + Honey treated group, indicating the recuperative effect of honey on liver bile ducts of the rats suffering from aflatoxicosis.

Increase in the levels of serum AST, ALT and GGT reflects loss of structural integrity of the liver. These enzymes are released into the bloodstream in the presence of hepatocellular degeneration and necrotic changes, resulting in, thereby, elevation of levels of serum AST, ALT and GGT [43]. In the current study, as shown in Table 2, while AF caused a significant elevation in the levels of AST, ALT and GGT in rats treated with AF, there were no significant differences in the levels of these enzymes between the AF + honey treated group and control group. Namely, administration of honey restored the increased activities of enzymes induced by AF to their control levels. These findings suggest that honey may have protective effects on the damages caused by AFB_1_, as evidenced by decreased AST, ALT and GGT levels.

Several studies indicate that AFB_1_ causes degenerative and necrotic changes in proximal tubular epithelial cells [44, 45] and hyaline casts [38] in the tubule lumen. Furthermore, enlargement of epithelial nuclei was demonstrated in an experimental aflatoxicosis study [26]. In the presented study, similar findings were observed in the AF-treated group. However, with the supplementation of honey these histological changes were significantly decreased in AF + honey-treated group. These histological results confirmed by biochemical findings. As a result, honey feeding protects the kidney against AF nephrotoxicity through reducing tubular damage, including tubular degeneration and necrosis, and MDA levels.

Membrane lipids are very sensitive to harmful effects of reactive oxygen species [46, 47]. The removal of one hydrogen atom is the initiating step in lipid peroxidation from an unsaturated fatty acid chains under the effect of ROS, which, in turn, result in an increase of MDA level [48, 49]. MDA is a widely used as an index of lipid peroxidation [50] and increased MDA content is an important indicator of oxidative membrane damage [51]. In the present study, MDA concentrations significantly increased in the erythrocyte, liver and kidney of rats treated with AF, which seem to be resulted from increased levels of ROS as a result of stress condition in the rats with AF intoxication. But honey treatment reversed the increased MDA contents to the control level, which is suggestive of that honey may be successful in quenching the free radicals, inhibiting lipid peroxidation and protecting membrane lipids from oxidative damage in liver of rats. The honey is shown to possess phytochemicals like flavonoids, effective in scavenging free radicals [52]. This might explain the reduction in MDA levels that we observed in the present study when honey was added to diet of the rats.

The increased activities of SOD, GR and CAT are known to serve as protective enzymes for elimination of reactive free radicals [53]. SOD catalyzes the dismutation of superoxide into oxygen and H_2_O_2_, and CAT is responsible for inactivating H_2_O_2_ into water and oxygen [54]. The finding in the present study showed that antioxidant enzyme activities such as SOD and CAT decreased significantly in the liver of the AF-exposed group, whereas the activities of these enzymes of AF + honey group were comparable with those of control group. The observation that application of honey caused the levels of SOD and CAT to increase to their normal levels in the AF + honey-treated rats indicates that the liver is restored to its normal activity by the protective action of honey. Honey possesses phytochemicals like flavanoids, which have been shown to be effective in scavenging free radicals [50]. This fact is further substantiated by the decrease in the levels of MDA upon honey administration. The ancillary enzyme GR activities in the erythrocytes of AF and AF + honey were higher than those in control group. Meanwhile, there were no significant differences in the activity of the drug metabolizing enzyme GST in tissues between the treatment groups. The reasons for such effects of honey are not understood at the present.

## Conclusions

It is concluded that honey with antioxidant properties appears to have a protective role in AF toxicity as it enhances the activities of liver function, decreasing level of MDA and the serum AST, ALT and GGT. The honey supplementation reduce the formation of the ROS or scavenges of these groups. Therefore, honey could has a potential rol as antioxidant for food supplementation or pharmaceutical industry. Further experiments should be performed to understand the underlying mechanism responsible for changing the balance between the oxidative processes and antioxidant defense systems, and serum marker enzymes.

## Abbreviations

AF, aflatoxin; AFB1, aflatoxin B1; ALT, alanin aminotransferase; AST,aspartate aminotransferase; CAT, catalase; GGT, gamma glutamil transpeptidase; GR, glutathione reductase; GSH, reduced glutathione; GST, glutathione-S-transferase; MDA, malondialdehyde; ROS, reactive oxygen species; SOD, super-oxide dismutase; TLC, thin layer chromatography
